# Obstructive Sleep Apnea and Coronary Artery Disease: From Pathophysiology to Clinical Implications

**DOI:** 10.1155/2013/768064

**Published:** 2013-04-15

**Authors:** Fernando De Torres-Alba, Daniele Gemma, Eduardo Armada-Romero, Juan Ramón Rey-Blas, Esteban López-de-Sá, José Luis López-Sendon

**Affiliations:** Acute Cardiac Care Unit, Department of Cardiology, La Paz University Hospital, Paseo de la Castellana 261, 28046 Madrid, Spain

## Abstract

Coronary artery disease (CAD) and obstructive sleep apnea (OSA) are both complex and significant clinical problems. The pathophysiological mechanisms that link OSA with CAD are complex and can influence the broad spectrum of conditions caused by CAD, from subclinical atherosclerosis to myocardial infarction. OSA remains a significant clinical problem among patients with CAD, and evidence suggesting its role as a risk factor for CAD is growing. Furthermore, increasing data support that CAD prognosis may be influenced by OSA and its treatment by continuous positive airway pressure (CPAP) therapy. However, stronger evidence is needed to definitely answer these questions. This paper focuses on the relationship between OSA and CAD from the pathophysiological effects of OSA in CAD, to the clinical implications of OSA and its treatment in CAD patients.

## 1. Introduction

Coronary artery disease (CAD) is a major health issue in developed countries and constitutes a significant cause of death and disability. The clinical spectrum of CAD ranges from stable angina pectoris to acute coronary syndromes (ACSs), a term which includes unstable angina (UA), non-ST elevation (non-Q wave) myocardial infarction (NSTEMI), and ST elevation myocardial infarction (STEMI) [1]. The primary pathologic process causing CAD is coronary atherosclerosis, which causes progressive coronary stenosis, provoking myocardial ischemia when myocardial oxygen demand exceeds oxygen supply, leading to angina pectoris. On the contrary, acute coronary syndromes are caused by the loss of integrity of the protective covering of some atherosclerotic plaques, leading to thrombus formation and subsequent vessel obstruction [2]. 

Despite the reduction in mortality rates that occurred in the past decades, it still affects 6.4% of adults in any of its forms and constitutes the cause of death of nearly 17% of adult population in the United States [3]. According to data from the Framingham Heart Study, a population-based longitudinal study, nearly one-half of males and one-third of females over 40 years of age will develop some manifestation of CAD [4]. 

OSA is a common disorder which has become an important public health problem, as it affects 2 to 7% of adults in the general population [5]. OSA is characterized by repetitive interruption of ventilation during sleep due to total collapse or narrowing of the pharyngeal airway despite breathing effort, resulting in a fall in oxygen saturation and arousal from sleep [6]. Repeated hypoxemia and arousals can lead to deleterious effects, ranging from daytime symptoms of disrupted sleep such as sleepiness, fatigue, or poor neurocognitive performance [7] to severe medical disorders [8]. A growing body of evidence links OSA with the development and progression of certain cardiovascular conditions such as systemic hypertension, pulmonary hypertension, CAD and myocardial ischemia, stroke, congestive heart failure, and cardiac arrhythmias [9]. 

This paper focuses on the relationship between OSA and CAD, from the pathophysiological effects of OSA in CAD, to the clinical implications of OSA and its treatment in CAD patients.

## 2. Pathophysiological Effects of OSA in CAD

The pathophysiological relation between OSA and CAD is complex, as many pathological changes induced by OSA in the cardiovascular system are involved and may interact to facilitate the development and/or progression of the pathological substrates of the different clinical conditions in the spectrum of CAD.

### 2.1. OSA and Coronary Atherosclerosis

The mechanisms leading to the formation and progression of atherosclerotic plaques involve a complex interaction between multiple factors, including oxidative stress, endothelial dysfunction, and inflammatory and immunologic factors [10]. 

Oxidative stress is enhanced in atherosclerosis, leading to endothelial damage and to oxidative modification of lipoproteins and other molecules [11]. This oxidized particles perpetuate endothelial damage [12] and promote accelerated accumulation of cholesterol in the atherosclerotic plaque [13]. In OSA patients, repeated hypoxia and reoxygenation during sleep can increase oxidative stress [10, 14–16], leading to vascular damage, and CPAP treatment reversion of these changes has been reported [17]. Enhanced lipid peroxidation resulting from this enhanced oxidative stress has also been observed in OSA patients [18], and in a murine model of chronic intermittent hypoxia combined with a high-cholesterol diet Savransky et al. [19] reported increased lipid peroxidation and development of atherosclerotic lesions. 

Endothelial dysfunction with loss of endothelium-derived nitric oxide production leads to impairment of the endothelium-dependent relaxation in the vasculature, and is an initial step in atherosclerosis [13]. This endothelium-dependent relaxation in other vascular beds is impaired in OSA patients [20–23] and CPAP treatment may improve these abnormalities [24].

 Endothelial dysfunction in OSA patients is independent from obesity and other traditional risk factors, as Namtvedt et al. have recently published [25]. A specific coronary endothelial function has been scarcely studied in patients with OSA. Kadohira et al. [26] reported a significant correlation between coronary endothelial dysfunction measured by vasoreactivity response to acetylcholine infusion and the severity of OSA. 

Inflammation plays also a major role in the development and progression of atherosclerosis. It has been repeatedly reported that OSA patients have increased serum levels of inflammatory substances [27–33] and adhesion molecules [14, 34, 35] and that chronic hypoxemia in OSA patients can activate specific inflammatory pathways [36, 37] that could facilitate the atherosclerotic process [38, 39]. CPAP treatment may also influence reversibility of these findings [40]. 

All these mechanisms facilitate the onset and progression of atherosclerosis, accelerating the process of arterial damage. Numerous studies have found a higher prevalence of atherosclerosis in vascular beds other than the coronary arteries, such as the carotid arteries [41, 42]. Similarly, Drager et al. found signs of atherosclerosis in large arteries of OSA patients without other risk factors for CAD, and the severity of the signs of atherosclerosis was correlated to the severity of OSA [43]. In another study by the same group, these signs of early atherosclerosis in OSA patients were responsive to CPAP treatment, supporting the concept that OSA may be an independent risk factor for atherosclerosis [44]. 

However, the evidence of more severe atherosclerosis specifically in the coronary vessels in OSA patients is scarcer. Recently Turmel et al. [45] studied 19 OSA patients with stable CAD who underwent three-dimensional intravascular ultrasound plaque evaluation and showed a higher atherosclerotic plaque volume in those patients with a more severe OSA. Similarly, Sharma et al. [46] found that the frequency of noncalcified or mixed plaques is much higher in patients with OSA than in non-OSA patients.

### 2.2. OSA and Thrombosis and Coagulation

The final pathogenic mechanism of an ACS is coronary artery thrombosis as a result of the loss of integrity of the covering of an atherosclerotic plaque, due to rupture or erosion, and subsequent platelet adhesion, activation, aggregation and thrombus formation [2]. 

Several studies have reported increased platelet activity in OSA patients [47], associated with oxygen desaturation [48] and reduced fibrinogen concentrations and fibrinolytic activity in OSA patients [49], among other abnormalities in the coagulation system [50]. These alterations have also been found to be reduced after CPAP treatment [51–53].

### 2.3. OSA and Myocardial Ischemia

Myocardial ischemia is the result of an imbalance between oxygen supply and demand in the myocardium. Some OSA-induced pathophysiological changes may have an influence in this equilibrium and provoke myocardial ischemia in some specific stressful situations. 

Airway obstruction during OSA can lead to recurrent high negative intrathoracic pressure that increases transmural gradients across the ventricles and thus myocardial afterload, leading to a rise in myocardial oxygen demand during the apneas, a fact that combined with the diminished oxygen supply can cause myocardial ischemia in some patients, such as patients with preexisting CAD [54]. Additionally, OSA leads to an increased sympathetic activity [55] due to hypoxia and hypercapnia, resulting in an increase in blood pressure and resting heart rate [56] that may induce myocardial ischemia [57]. 

Clinical evidence of myocardial ischemia induced by OSA has been addressed in several studies. Symptoms in some forms of ACS, such as nocturnal angina, can be triggered by OSA [58]. Similarly, nocturnal ST-segment depression is observed among patients with known CAD and OSA [59] but is also reported in patients with OSA without known CAD [60, 61]. CPAP is able to improve these abnormalities [54]. 

However, whether severe OSA is a trigger of nocturnal myocardial injury in patients with CAD is under discussion. In a prospective study of 15 patients with known CAD and moderate-to-severe OSA (apnea/hypopnea index (AHI) > 30), Gami et al. [62] reported no evidence of myocardial injury assessed by a third-generation troponin T assay during sleep. However, in a more recent study by Randby et al. [63] in a community-based sample of patients without known CAD, high-sensitivity cardiac troponin T levels were elevated in 53% of patients with OSA. This high prevalence lost significance after adjusting for other significant risk factor resulting in absence of association between high-sensitivity cardiac troponin T levels and OSA severity. The high-sensitivity cardiac troponin T detection among those patients may have been probably related to the higher prevalence and grouping of cardiovascular risk factors among patients with OSA, rather than to a nocturnal pattern of myocardial injury in patients without CAD.

In patients with an ACS, acute ischemia of a region of the myocardium is caused by the complete or partial cessation of coronary blood flow by a thrombotic occlusion of a coronary vessel, leading to a wide range of clinical manifestations, from unstable chest pain to sudden cardiac arrest. OSA may influence the onset of acute ischemic events, as ACS [64] and sudden cardiac death [65] are more frequent during nighttime in OSA patients, contrarily to the usual diurnal pattern, although there is not enough evidence to conclude that OSA can be a definite trigger of ACS, even in those occurring at night. 

However, some authors state that intermittent hypoxia that occurs at night in OSA patients may play a role in the development of ischemic preconditioning in the myocardium and is thus protective against myocardial ischemic insults by the regulation of critical transcription factors that play important roles in coronary endothelium [66]. Mechanisms underlying ischemic preconditioning are under active research, and clinical translation of the growing body of basic research is still to come [67]. In a recent observational study by Shah et al. [68], infarct size measured by peak troponin levels was assessed in 136 patients after an AMI who were screened for OSA. Troponin levels after an acute nonfatal AMI were significantly lower in patients with a more severe SAHS, after adjusting for other cardiovascular risk factors. This may be due to ischemic preconditioning in the myocardium, although more studies are needed to confirm this hypothesis and to better understand the mechanisms responsible for this response. Recently, a study by Berger et al. in patients presenting with AMI showed that those patients with sleep disordered breathing showed a greater mobilization of endothelial progenitor cells and increased vascular endothelial growth factor expression compared to patients with normal breathing [69].

Similarly, another possible adaptive mechanism of coronary circulation to chronic intermittent ischemia of OSA patients is the development of coronary collaterals. Coronary collateral formation from healthy vessels to an ischemic region of the myocardium is a well-known phenomenon in patients with chronic ischemic heart disease, present in 50% of patients with severe obstructive coronary lesions or totally occluded coronary arteries that can provide a perfusion reserve in case of increased myocardial oxygen demand in the affected territory. Mechanisms and factors associated with the development of these collaterals are not totally understood, and chronic intermittent hypoxia in OSA patients may play a role in this scenario, via the upregulation of critical vascular growth factors involved in vessel proliferation [70, 71]. In fact, Steiner et al. [72] reported a significantly higher coronary collateral vessel development in patients of totally occluded coronary arteries and OSA compared to those patients without OSA, suggesting that chronic intermittent hypoxia of patients with OSA may be a significant factor affecting growth of CCVs as a compensatory mechanism to chronic myocardial ischemia. However, more studies are needed to better understand the underlying mechanisms of the adaptive responses of coronary circulation to chronic intermittent hypoxia and the clinical implications of these responses in OSA patients. 

## 3. Clinical Implications of OSA in Patients with CAD

### 3.1. Epidemiology

The prevalence of OSA in patients with CAD is higher as in normal population, as numerous studies state [73–80]. Mooe et al. [73] reported a prevalence of OSA (AHI > 10) of 37% in 142 patients with stable angina pectoris and angiographically confirmed CAD, and Peker et al. [74] reported a prevalence of OSA of 30% in those patients admitted with an ACS. A more recent report from Konecny et al. [75] highlights the underrecognition of OSA among patients admitted for an ACS, with a prevalence of OSA of 69% and severe OSA (AHI > 30) of 34%.

Other studies reported a wide range of prevalence, ranging from 26% to 66% [76–80], partially explained by the different value of AHI used to establish the diagnosis of OSA in the different studies. 

However, despite the high prevalence described in some of these studies, the current guidelines of practice guidelines for the management of STEMI [81] and NSTEMI and UA [82] do not state any recommendation for screening OSA in patients presenting with an ACS.

### 3.2. OSA as a Risk Factor for CAD

There is a growing body of literature addressing the role of OSA as an independent risk factor of CAD [83]. As both disorders share common risk factors, such as obesity, male sex, age, and smoking, overlapping without a causal relationship may be possible [7, 84, 85]. Moreover, several of well-established risk factors for CAD have been associated with OSA in multiple studies, specially systemic hypertension [28, 86, 87], but also diabetes mellitus [88, 89], dyslipidemia [90], and metabolic syndrome [91]. Therefore, proving that OSA can independently cause CAD remains a difficult issue. 

OSA is independently associated with subclinical coronary atherosclerosis measured by coronary calcification in computed tomography [92], and there is also a higher prevalence of noncalcified obstructive atherosclerotic plaques in OSA patients, as stated above. Several clinic-based longitudinal studies have found an association between OSA and the development of CAD, after adjusting for other risk factors, mainly in untreated individuals referred to CPAP treatment [93–97]. Data of more than 6000 patients from the community-based Sleep Heart Health Study found an association between OSA and incident CAD in those patients with the most severe OSA [98], but a more recent analysis of the same cohort, with longitudinal data after a 8.7-year followup, found no association of OSA with incident CAD, after adjusting for other risk factors, except in patients younger than 70 years of age, in which the risk of CAD was slightly increased and was also slightly higher in patients with the most severe OSA [99]. On the contrary, in an other recent observational study of >1000 patients, Shah et al. [100] found a significant association between OSA and incident coronary events or cardiac death after adjusting for other traditional risk factors. Similarly, García-Río et al. [80] reported significant association between OSA and acute myocardial infarction. Although most of these observational data suggest a strong association, data are not conclusive and evidence is still insufficient to establish a causative role of OSA in the development of CAD. 

### 3.3. OSA and Outcomes of CAD

OSA may also influence outcomes when present in patients with CAD [101, 102], but its effect remains a controversial issue as studies have yielded discrepant data. In a prospective cohort study of >400 patients with stable angina pectoris and CAD confirmed by coronary angiography, with a followup of >5 years, OSA was associated with a higher rate of death, myocardial infarction, and stroke [103]. More recent long-term follow-up data from the same group [104] show a significant association of OSA and stroke among stable CAD patients. Similarly to chronic CAD patients, patients who present with an ACS as the first manifestation of CAD also show worse outcomes. Yumino et al. [105], in a prospective cohort study of 89 patients with ACS who underwent polysomnography found OSA in 57% of patients. After a mean followup of >7 months, the incidence of cardiac death, reinfarction or new revascularization was higher in patients with OSA compared to those without OSA. Similarly, a more recent study by Lee et al. [78] showed a higher incidence of major adverse events patients with severe OSA after a STEMI. Non-STEMI patients with OSA have also been reported to have poorer in-hospital outcomes [106]. Recovery of the left ventricle systolic function after an acute myocardial infarction is one of the strongest long-term prognostic factors. There are data that suggest that OSA may inhibit the recovery of the left ventricular systolic function after an acute myocardial infarction [107]. However, other studies did not find an association between OSA and recurrent cardiovascular events in CAD patients [108].

Data suggest that OSA may worsen prognosis in all the spectrum of CAD patients, but more research is necessary in order to determine whether diagnosing and treating OSA may influence the prognosis of patients with CAD.

### 3.4. Treatment of OSA and CAD

CPAP continues to be the primary therapy for patients with symptomatic OSA. Most studies report that the treatment of OSA with CPAP results in a favorable clinical effect on various factors associated with the structure and function of the cardiovascular system, like reduction of sympathetic activity, improved vasodilatadory capacity of the endothelium, hypercoagulability and systemic inflammation, increased intrathoracic pressure, and other effects that lead to a reduction in myocardial oxygen demand and increased coronary blow flow [17, 24, 40, 51–54].

Treatment of OSA with CPAP in symptomatic patients without known CAD has been associated with a reduction in cardiovascular events [96, 97, 109]. However, the question whether CPAP treatment in nonsleepy OSA patients aiming at a decrease in cardiovascular events still remains unclear. CPAP treatment did not significantly reduce the incidence of cardiovascular events compared to no intervention in general population, as data from a recent multicenter randomized controlled clinical trial show [110]. However, adherence to treatment had a significant impact in the results of this trial, as when patients who were adherent to CPAP treatment more than 4 hours per day were analyzed a statistically significant reduction in the incidence of CV events was found. This is consistent with other observational studies [111, 112] that support the hypothesis of a protective effect of CPAP treatment in OSA patients regardless of sleepiness symptoms.

However, information regarding this question in the specific population of patients with CAD is more scarce and derived only from observational studies, and thus, the impact of the OSA treatment as a secondary prevention measure for CAD patients intended to reduce cardiovascular events in the long term remains unclear, mainly due to the lack of a placebo-controlled, randomized treatment trials in patients with OSA and CAD. 

In 54 patients with chronic stable CAD and severe OSA, an observational study by Milleron et al. [95] with a median followup of 86 months reported a beneficial effect of CPAP treatment, in terms of a reduction and a later occurrence of recurrent cardiovascular events. However, Cassar et al. [113] reported fewer cardiac deaths but not fewer cardiovascular events in CPAP-treated OSA patients undergoing percutaneous coronary intervention for stable angina. In a recent observational study by García-Río et al. [80], acute myocardial infarction patients with untreated OSA had a higher rate of recurrent myocardial infarction and necessity of new revascularization compared to treated OSA patients and non-OSA patients. In this scenario, adherence to CPAP treatment in patients without daytime sleepiness may be a concern, as patients with CAD, especially after an ACS, have shown a very low adherence to behavioral or lifestyle preventive measures, in contrast to a high adherence to pharmacological preventive measures [114]. However, a study by Sampol et al. [115] has shown that adherence to CPAP treatment in patients with CAD is not influenced by the absence of sleepiness symptoms. 

However, although the above data suggest a possible benefit of CPAP treatment in this population, they come from observational studies, and randomized clinical trials are needed to establish whether CPAP treatment should be recommended to chronic or acute CAD patients without daytime sleepiness. This issue is addressed by three ongoing randomized clinical trials from which results are strongly awaited. The first randomized trial (Continuous Positive Airway Pressure Treatment of Obstructive Sleep Apnea to Prevent Cardiovascular Disease—SAVE Trial, NCT00738179) aims to test whether long-term use of CPAP can reduce the incidence of cardiovascular events (cardiovascular death, nonfatal AMI, nonfatal stroke, hospital admission for heart failure, and new hospitalization for unstable angina or transient ischemic attack) in 5000 patients with evidence of established coronary or cerebrovascular disease over a mean follow-up period of 4 years. The second trial (The Randomized Intervention With CPAP in Coronary Artery Disease and Sleep Apnea—RICCADSA Trial, NCT0051959) addresses this issue in stable CAD patients. This randomized trial that is expected to complete recruitment by December 2012 aims to include more than 500 patients with CAD undergoing planned percutaneous or surgical revascularization, and address if CPAP treatment reduces the combined rate of new revascularization, myocardial infarction, stroke, and cardiovascular mortality over a mean follow-up period of 3 years in CAD patients with OSA and without daytime sleepiness [116]. The third trial (Continuous Positive Airway Pressure in Patients With Acute Coronary Syndrome and Obstructive Sleep Apnea—ISAACC trial, NCT 01335087) addresses this issue in the setting of the acute coronary syndromes. This randomized trial will include more than 1800 patients with a recent ACS and will assess whether CPAP treatment will reduce the rate of major cardiovascular events (cardiovascular death, nonfatal AMI, nonfatal stroke, hospital admission for heart failure, and new hospitalizations for unstable angina or transient ischemic attack) in patients with non-ST elevation or ST elevation ACS admitted in a coronary care unit and co-occurring with OSA. These trials will probably be the pivotal trials which will definitely answer the question of whether CPAP treatment influences prognosis of CAD patients with OSA both in the acute and in the chronic settings. 

## 4. Summary

CAD is a complex disease with a wide range of clinical manifestations. OSA is as well a condition with numerous systemic implications, which can influence CAD in numerous phases and pathological processes. Moreover, despite that CAD and OSA share risk factors and that systemic conditions influenced by OSA have a role as well in the pathogenesis of CAD, evidence is lacking to address OSA as a risk factor of CAD, as a prognostic factor of CAD. Similarly, more evidence is needed to address CPAP treatment as a useful therapy for patients with CAD and nonsymptomatic OSA. However, research in this field is growing rapidly, and data answering some of these questions may soon, be available.
